# Small Molecule Optical Probes for Detection of H_2_S in Water Samples: A Review

**DOI:** 10.1021/acsomega.3c08573

**Published:** 2024-03-21

**Authors:** Ranjana M, Rashmi M. Kulkarni, Dhanya Sunil

**Affiliations:** Department of Chemistry, Manipal Institute of Technology, Manipal Academy of Higher Education, Manipal, Karnataka, India 576104

## Abstract

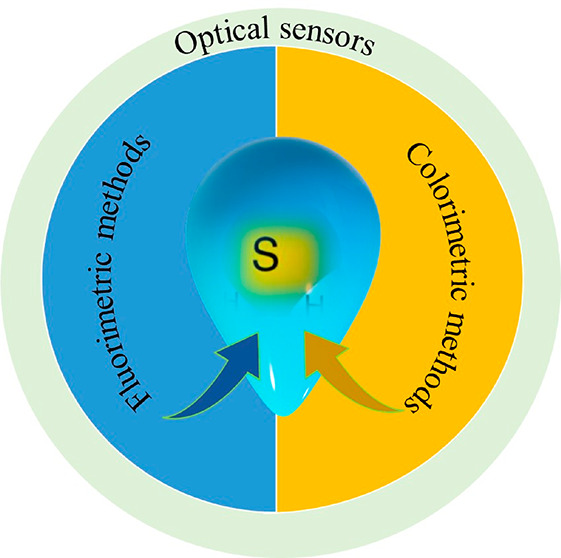

Hydrogen sulfide (H_2_S) is closely linked to not only environmental hazards, but also it affects human health due to its toxic nature and the exposure risks associated with several occupational settings. Therefore, detection of this pollutant in water sources has garnered immense importance in the analytical research arena. Several research groups have devoted great efforts to explore the selective as well as sensitive methods to detect H_2_S concentrations in water. Recent studies describe different strategies for sensing this ubiquitous gas in real-life water samples. Though many of the designed and developed H_2_S detection approaches based on the use of organic small molecules facilitate qualitative/quantitative detection of the toxic contaminant in water, optical detection has been acknowledged as one of the best, attributed to the simple, highly sensitive, selective, and good repeatability features of the technique. Therefore, this review is an attempt to offer a general perspective of easy-to-use and fast response optical detection techniques for H_2_S, fluorimetry and colorimetry, over a wide variety of other instrumental platforms. The review affords a concise summary of the various design strategies adopted by various researchers in constructing small organic molecules as H_2_S sensors and offers insight into their mechanistic pathways. Moreover, it collates the salient aspects of optical detection techniques and highlights the future scope for prospective exploration in this field based on the limitations of the existing H_2_S probes.

## 1 Introduction

Hydrogen sulfide (H_2_S) is a member of the reactive sulfur species family and is colorless, flammable, water-soluble, corrosive, and acutely toxic with a characteristic “rotten egg” smell. The H_2_S present in raw waters originates from mostly natural sources and industrial activities. The gas can be found naturally as hydrosulfide (H_2_S) or monohydrogen sulfide (HS^–^) in rivers and waste waters; volcanic activity, natural gas, and crude oil being the other natural sources, in addition to the anaerobic activity of the sulfate-reducing bacteria.^1^ H_2_S can be generated during industrial processes through protonation of HS^–^ and sulfide (S^2–^), which are extensively used to produce sulfur, sulfuric acid, dyes, detergents, cosmetics, etc. and moreover through the oxidation/degradation/disintegration of sulfur-rich amino acid residues in meat proteins, as well as several organic compounds produced in paper, petrochemical treatment, biogas production, printing, mining, leather manufacturing, dyeing industries, and sewage treatment plants.^2−8^ Large amounts of sulfur containing wastewater are generated due to the rapid advancements in industries.

The impact of the smallest bioactive thiol H_2_S on the environment and human health has severe consequences. The chalcogen hydride gas induces contamination of the surrounding environment causing acid rain, corrosion of metallic structures, and leaching of heavy metals.^9,10^ Further, the inappropriate disposal of industrial wastewater that contains H_2_S into the environment without suitable treatment processes and the sulfides that reduce the dissolved oxygen concentration in surface waters negatively influence aquatic organisms as well as the metabolism of sulfate-reducing bacteria.^11^ Moreover, the small molecule has garnered extensive attention as a gasotransmitter, a role similar to that of other well-known signaling molecules like nitric oxide and carbon monoxide. At lower concentrations, H_2_S plays a pivotal role in regulating various physiological functions including vasodilation, neurotransmission, anti-inflammatory effects, and cellular respiration at lower concentrations. However, the gas when present at higher concentrations has a direct impact on human health. The toxicity profile of the gas is analogous to that of carbon monoxide, as it binds with iron in the mitochondrial cytochrome enzymes, inhibiting cellular respiration. It can pose severe physiological and biochemical problems including chronic diseases of blood, eyes, digestive, nervous, and respiratory systems.^12−17^ Exposure to high concentrations (biologically relevant levels of H_2_S vary from nanomolar to micromolar levels)^18^ of the toxic gas can result in health risks including Alzheimer’s disease, diabetes, and cardiovascular effects.^19,20^

H_2_S dissociates to form HS^–^ and S^2–^ ions upon hydrolysis in water.^15,21−23^ The pH is a deciding factor for the presence of relative concentrations of these species in water, with H_2_S concentrations rising with dropping pH. Mostly HS^–^ exists in water at pH 7.4, whereas about one-third exists as undissociated H_2_S and S^2–^ in appreciable concentrations above pH 10. Further, sulfate can be microbially reduced to sulfide in anaerobic water.^24^ The World Health Organization has estimated the taste and odor thresholds for H_2_S in water to be 0.05–0.1 mg L^–1^, whereas the maximum allowable S^2–^ level in drinking water is defined as 15 μM.^25^ The presence of H_2_S above the permitted levels is a significant pollution indicator, and therefore, its quantitative detection is quite essential, particularly in susceptible occupational settings.^26^ As H_2_S is widely used in the production of essential marketed products including medicines, cosmetics, dyes, pesticides, etc. and is formed frequently as a byproduct in various industrial processes, its emissions and subsequent pollution are inevitable.^27−31^ This issue highlights a pressing demand for instant, sensitive, and selective detection technologies for H_2_S.

## 2 Techniques for Detecting H_2_S in Water Samples

The ever-growing concern about the ecological effects caused by industry effluents has driven the need for implementing robust monitoring approaches that assist the compositional evaluation of the discharges before they are released to the environment. Quantification of H_2_S/S^2–^ in drinking and river water is very critical because their levels beyond the threshold limit value is found to be toxic to both aquatic and human health.^32,33^ Therefore, several traditional strategies to detect H_2_S in water samples including iodometry, methylene blue, colorimetry, fluorimetry, mass spectrometry,^34,35^ metal induced sulfide precipitation,^36−40^ as well as electrochemical^41−43^ and chromatography (GC and HPLC) techniques have emerged. Sophisticated techniques such as electrogenerated chemiluminescence^44−46^ and inductively coupled plasma-atomic emission spectrometry^34^ are also reported for the recognition of H_2_S, which often require expensive instrumentation and tiresome analysis. Though most of these strategies designed to aid H_2_S detection are viable across various instrumental platforms, distinct advantages including simplicity, sensitivity, selectivity, low-cost, and rapid tracking applicability of H_2_S in environmental samples^47−52^ using small molecule optical sensors (both fluorimetric and colorimetric) have received substantial research interest.

The present review attempts to provide a broader perception of easy-to-use, selective, and sensitive optical detection techniques: colorimetric and fluorometric sensing for H_2_S that enables a fast response time in environmental water samples. Besides, the article affords a concise summary of small organic molecule H_2_S sensors reported by various researchers and collates their salient aspects. The different sensing mechanisms of these H_2_S sensing chromophores/fluorophores are illustrated. Further, based on the limitations of these probes, the future scope of exploration in this field is also discussed. The supporting tables and pictorial representations in this review facilitate easy understanding.

## 3 Small Molecules as Fluorimetric Probes

With a glimpse into the history of fluorescent organic probes, quinine sulfate stands out as the initial fluorescent organic molecule, credited to Sir John Herschel in 1845.^53^ However, it is worth noting that the term “fluorescence” was not introduced until 1852 when George Stokes published an extensive article on fluorescence.^54^ Despite this, the blue fluorescence emitted by quinine sulfate under ultraviolet (UV) light paved the way for the exploration of numerous organic compounds with fluorescence properties.^55^ Over time, a diverse array of organic molecules capable of fluorescing in different colors have been discovered or synthesized. Two notable examples are fluorescein^56^ and rhodamine^57^ derivatives, initially reported in the late 19th century. These compounds have gained significance as representative platforms extensively utilized in contemporary fluorescent labels and probes for bioimaging applications. Additionally, BODIPY dyes^58^ and cyanine dyes^59^ are frequently employed in the development of bioimaging tools due to their remarkable fluorescence properties. The continuous exploration and utilization of such organic molecules have significantly contributed to the advancement of fluorescent technologies in various scientific and medical applications.

Fluorescence (FL) detection is a widely used analytical tool for optical sensing of H_2_S, attributed to its low cost, simple instrumentation, operational ease, easy testing process, real-time rapid detectability, good sensitivity, and selectivity.^60,61^ FL signals can be assessed in various ways including direct (turn-off/turn-on FL), ratiometric (variation in FL intensities at two different emission wavelengths), and energy transfer methods.^62−64^

Further, fluorophores can be embedded on solid supports to obtain solid-state sensors, which can be more effective compared to solution phase-based probes because of their portable and low-cost nature.^26,65−67^ Several paper-based sensors based on FL responses that can facilitate not only rapid response and simple operation but also enable reversible and reproducible low-cost testing of H_2_S in water samples are reported, with some showing colorimetric responses alongside. Moreover, these paper sensors, which can either show turn-on or turn-off FL signals can be coupled with image processing and analysis software installed in smartphones to realize qualitative as well as quantitative detection of H_2_S. These FL-based solid sensors facilitate H_2_S detection for real-time practical applications including in situ field testing.^68−73^

### 3.1 Design Strategy for Fluorimetric Probes

Generally, the small molecule sensor comprises a fluorophoric unit and a H_2_S recognition site in its structural design. The fluorescent probes thus constructed for detecting H_2_S in water samples should meet the following requirements: (i) notable changes, either turn on, turn off, enhancement, or ratiometric FL signal, (ii) good photostability, (iii) high FL quantum yield (QY), (iv) fast FL response, (v) good selectivity and sensitivity, (vi) low detection limit (LOD) value, and (vii) water solubility. The H_2_S recognition site and the respective sensing mechanism of the probe can be confirmed not only by various spectroscopic techniques including ^1^H NMR, ESI mass, and UV titration but also through theoretical studies. The fluorescent probes reported for monitoring H_2_S levels in water samples can be categorized based on their different reaction mechanisms: (i) reduction of azides (-N_3_) or hydroxyl amines (-NHOH) or nitro (NO_2_) groups to amino (NH_2_) groups,^74−77^ (ii) reduction of selenoxide to selenide,^78−81^ (iii) binding affinity toward copper ions resulting in copper sulfide (CuS) precipitation,^82,83^ (iv) ligand exchange with metal complexes,^28,84−91^ and (v) nucleophilic addition of H_2_S^92−94^ including dual nucleophilic, Michael addition, and double bond addition reactions, as well as thiolysis of leaving groups (dinitrophenyl (DNP) ether,^95−99^ dinitrobenzenesulfonate (DNBS) ester, and electrophilic cyanate (-CN)).^100−104^

**Figure 1:**
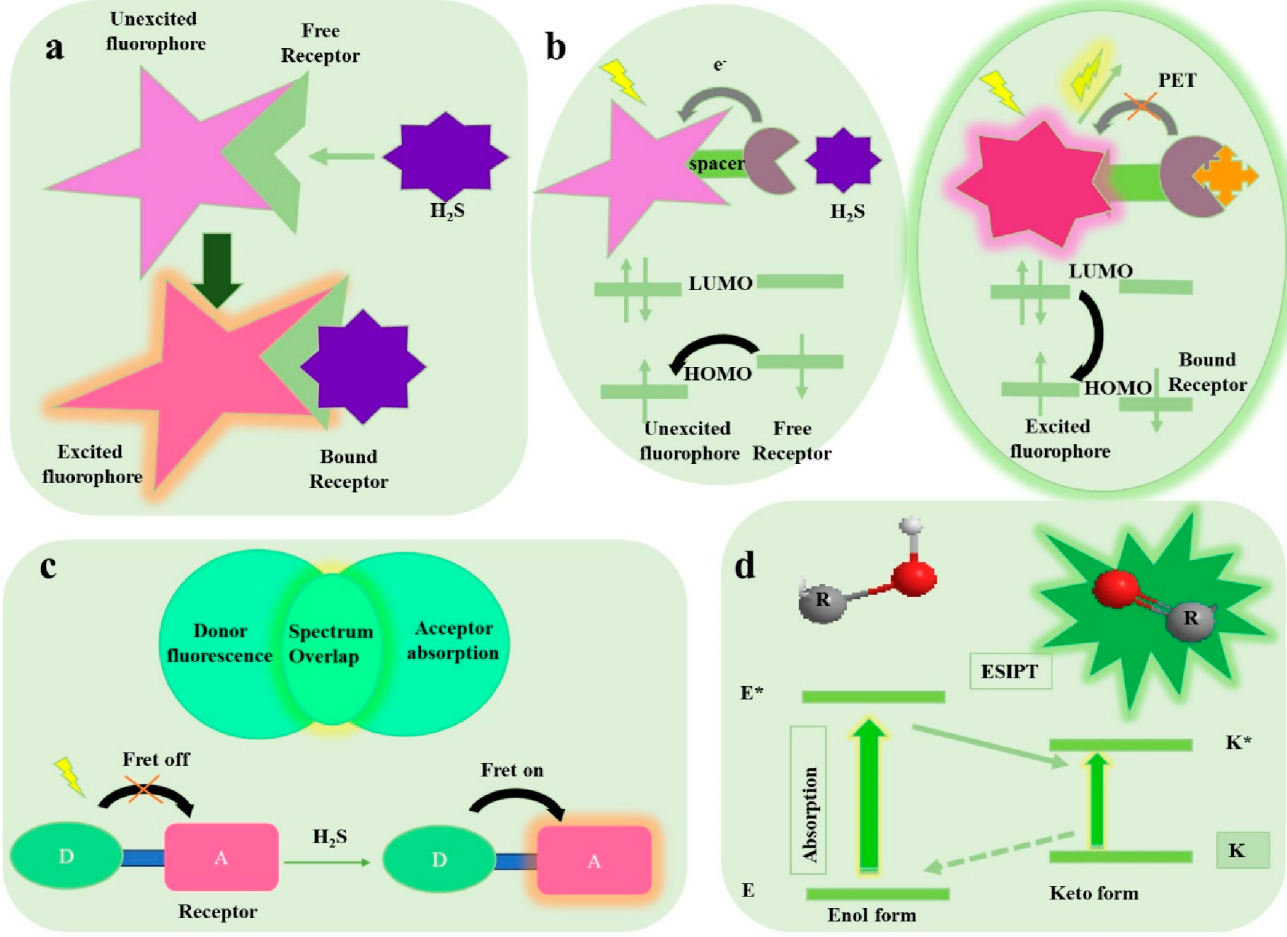
Schematic representation of (a) ICT, (b) PET, (c) FRET, and (d) ESIPT phenomenon in H_2_S sensors.

The different photophysical FL transduction mechanisms including intramolecular charge transfer (ICT), photo induced electron transfer (PET), fluorescence resonance energy transfer (FRET), and excited-state intramolecular proton transfer (ESIPT) contribute to the variations in electron transfer upon H_2_S binding to the small molecule probe as portrayed in Figure 1. In ICT, the absorption and emissions are significantly shifted within the molecule due to charge transfer from the electron donor (D) to acceptor (A), which are conjugated without a spacer within the small molecule (Figure 1a). This process happens when the electrical structure of a molecule is altered, usually leading to a redistribution of the electron density inside the molecule. An electron may move from one area of the molecule to another, or charge-separated states may be formed by this redistribution. The design of a PET H_2_S probe includes a receptor with a nonbonded electron pair and a fluorophore separated by a short aliphatic spacer unit.^105^ In the unbound state, an intramolecular electron transfer occurs from the HOMO of the receptor to the LUMO of the excited fluorophore, as shown in Figure 1b. The bound receptor coordinates to H_2_S through the electron pair, making the receptor HOMO lower than that of the fluorophore, switching off the PET and turning on the FL. The FL responses perceived during H_2_S recognition are different, as PET quenching does not result in any emission band shifts, whereas ICT sensors show a ratiometric response with vivid spectral shifts. In FRET as depicted in Figure 1c, a transfer of excitation energy occurs due to the interaction between D and A, which is influenced by various factors including the D–A distance, the D–A spectral overlap, the dipole moment of the molecular system etc. ESIPT usually occurs in 5- or 6-membered ring bearing small molecule (Figure 1d) fluorogens that can avoid the inner filter effect or self-absorption to enhance the FL performance.^48^ The unexcited molecules that exist in intramolecular hydrogen (H)-bonded enol (E) form experiences a rapid tautomerization into its keto form (E* → K*) upon photo excitation and associated emission changes. The ESIPT process occurs to stabilize the keto form through intramolecular H-bonds. The K* form relaxes to the ground K state and exhibits FL very often as the ESIPT process is significantly faster than the radiative decay. Moreover, the substantial differences in the absorbing (E*) and emitting (K*) species generate a large FL Stokes shift for improved FL analysis. A suitable fluorophore platform and the recognition moiety are thus crucial for fluorescent probes for effective detection of H_2_S in water samples.

### 3.2 Fluorimetric Sensors Based on Various Reaction Mechanisms

The small molecular probes reported for detecting H_2_S in water samples along with their respective sensing mechanisms are reviewed below.

#### 3.2.1 Deprotonation Hindering ICT

“Salen-type” ligands are a class of coordination compounds produced when a bisaldehyde condenses with a diamine. The introduction of large electronegative oxygen atoms to the C=N–group of salen-type ligands results in salamo-type probes with improved coordination flexibility, structural stability, high selectivity, and sensitivity.^106−113^ Based on this background, Guo et al. prepared a rigid structured fluorescent probe **1** with naphthol units and π-conjugation for dual-channel detection of H_2_S.^114^ The sensor worked in a wide pH range of 4–9 with a response time of <3 s. The deprotonation of the hydroxyl groups hindered the ICT in the probe in the presence of H_2_S, which resulted in vivid changes in the FL intensity, facilitating the detection of the biothiol. Further, as azo-dye containing Schiff bases are known for their excellent chromophoric strength, Manna et al. prepared an azo-dye based bis-Schiff base probe **2** that could detect S^2–^ ion both visually and spectrophotometrically in pure aqueous medium, within 5–10 pH through a deprotonation mechanism.^115^ The probe displayed good water solubility due to the -SO_3_H group and high binding affinity with low LOD due to the presence of two D and two A sites compared to a single D/A-based sensor. The probe was weakly fluorescent because of a nonradiative decay process in the excited state that originated by the combination of PET from the -OH moiety and ESIPT to the benzil dihydrazone fluorophore. However, the basic S^2–^ ions induced easy deprotonation of the hydroxyl moiety extending the electron delocalization to inhibit the ESIPT process. Besides, the improved electron density on the oxygen atom transferred to the electron withdrawing azo unit via ICT to inhibit PET improved the FL emission. The reversible and hence reusable probe showed a sulfide ion triggered visually observable color transition from yellow to deep orange with potential application in real samples and in developing molecular logic gates.

#### 3.2.2 Thiolysis of Leaving (DNP, NBD, DNBS, CN) Groups

H_2_S induced thiolysis and subsequent cleavage of leaving groups such as DNP, 7-nitro-1,2,3-benzoxadiazole (NBD), DNBS, and CN^–^ groups to transform a nonfluorescent probe to a fluorophoric product are a well-established sensing mechanism. Few optical sensors based on the thiolysis reaction present both colorimetric and fluorometric responses for fast detection of S^2–^.^116,117^ Zhong et al. synthesized a D-π-A structured probe **3** incorporating a highly photostable and fluorescent 4-diethylaminosalicylyl core appended with 4-diethylamino (D) and 1,4-dimethylpyridinium iodide (A) groups to realize an ICT favored emission mechanism.^118^ The DNP segment served as both the FL quenching and H_2_S recognition site through the D-excited PET process. The HS^–^ induced thiolysis of DNP ether to release the fluorophore prevented D-PET to recover the ICT and facilitate a FL turn on H_2_S detection mode in real water samples.

Recently, near-infrared (NIR) fluorescent probes that display low background interference have been constructed for the sensitive identification of H_2_S, while they feature comparatively longer response times (around 30 min), hampering rapid sensing. However, Jin et al. could develop a dicyanomethylene-4*H*-chromene based NIR probe **4** that could detect H_2_S in real water samples within 3 min.^119^ The probe doped test strips and nanofibrous films demonstrated excellent prospects for on-site as well as real-time detection of H_2_S in environmental samples. Further, Zhong et al. developed a colorimetric and NIR fluorescent probe **5** incorporating diethylamino (D) and 2-(3-cyano-4,5,5-trimethylfuran-2(5*H*)-ylidene) malononitrile (A) units.^120^ The typical D-π-A structured fluorophore showed an ICT enabled red-shifted emission in the NIR region. The DNP ether served as both the FL quencher and the H_2_S recognition site through donor-excited D-PET and ICT blocking mechanisms. The HS^–^ triggered thiolysis of DNP ether hindered the D-PET to recover the ICT, which provided a turn on FL response. Moreover, the colorimetric detection was possible by visualizing the H_2_S induced transformation of a colorless probe to bluish-purple.

Ma et al. constructed two 4-hydroxy-1,8-naphthalimide based fluorescent sensors **6a** and **6b** with DNP ether as the H_2_S responsive site for optimal functioning in 5–8 pH range.^121^ Though both the probes displayed a >30-fold increased FL response, the dual site probe **6b** was found to be superior for quantitative detection due to a wider linear range between FL intensity and H_2_S concentration compared to the single recognition site probe **6a**. Liu and Feng reported a visible light excitable 3-hydroxyflavone-based ESIPT probe **7** with high FL QY to rapidly detect H_2_S in aqueous solution.^122^ The thiolysis of DNP ether displayed a color change from pale yellow to deep yellow and turned on the ESIPT for sensing H_2_S. The probe revealed many advantages including easy synthesis, visible light excitability, rapid detection within few minutes, dual colorimetric and fluorimetric responses, and high selectivity as well as sensitivity toward H_2_S.

The double nucleophilic character of H_2_S enables the substitution of the DNP moiety of a FL probe by H_2_S-facilitated nucleophilic addition and further thiolysis to generate the fluorophore. Besides, previous literature reports suggest that incorporation of an aldehyde unit into the ortho-position of the DNP unit could improve the selectivity as well as accelerated response toward H_2_S.^123−126^ Centered on these evidence, Gao et al. reported a rhodol derivative **8** anchored with a DNP moiety to bring in the FL quenching effect.^40^ The nucleophilic H_2_S addition with the aldehyde group located at the ortho position of the DNP induced intramolecular thiolysis of the ether to free the fluorophore for a rapid FL turn-on response. The H_2_S induced transformation from colorless to pink solution is an add-on feature to detect the thiol by the naked eye.

NBD is an attractive leaving moiety for thiolysis mediated S^2–^ detection^127^ because of its long wavelength emission and good water-solubility.^128−134^ Better selectivity and sensitivity endow FRET based sensors with superiority over turn-off, turn-on, and ICT based probes.^134,135^ Therefore, Sureshkumar et al. incorporated a NBD unit amine into a piperazine appended naphthalimide scaffold to construct a FRET sensor **9**.^51^ The NBD quenched the donor fluorescence by FRET, and subsequent S^2–^ induced thiolysis released the FRET. The development of a pink color in the paper test strips enabled monitoring of S^2–^ levels in environmental water samples. The low LOD of the fluorimetric sensor could be further utilized to check the drinking water quality after its purification and supply to the public. Yang et al. developed a ratiometric fluorescent probe **10** based on acridone (D) and NBD (A) with piperazine as the linker to construct yet another FRET based sensing platform.^136^ Probe **10** with NBD-piperazine as the recognition site exhibited a significant enhancement in the yellow to blue FL emission ratio under UV light in combination with orange to pink visual color change. Further Kim et al. constructed probe **11** to detect S^2–^ in various water samples through FL quenching from bright yellowish green to weak blue and colorimetric change from orange to pink.^137^ The probe coated test strips were applied for the on-site quantification of S^2–^.

In consensus with the importance for environmental monitoring, Jin et al. demonstrated a FL turn-on probe **12** carrying an electrophilic cyanate moiety that poses a small steric hindrance as the H_2_S reaction site.^138^ A fast nucleophilic reaction between the cyanate group and the analyte thiol facilitated a quick response within 5 min with a yellow to brownish red color change coupled with enhancement in NIR fluorescence. Thiolysis of DNBS has many advantages including a single-step reaction to attach DNBS to the central core, nitro group induced reduction in optical signals, and high selectivity of DNBS toward H_2_S than other competitive thiols, such as cysteine and glutathione. Therefore, Xie et al. prepared a NIR fluorescent probe **13** with a conjugated D-A-D framework by incorporating DNBS as H_2_S recognizing unit to benzothiadiazole and thiophenes as the electron A and D units.^48^ The cleavage of the sulfonamide group released the D-A-D group turning on the NIR FL response in the presence of H_2_S. The test strips dipped in the probe solution could be used to visually monitor the orange to purple-red color change in addition to the orange to purple FL responses in the presence of the biothiol. Among the two turn-on fluorescent probes **14a** and **14b** containing benzothiazole and DNBS groups reported by Liu et al, **14b** was found to be more water-soluble due to its positive charge, whereas **14a** exhibited a fast response to H_2_S with better selectivity.^52^ H_2_S induced DNBS cleavage to form a fluorophore with hindered PET led to a great increase in FL intensity.

#### 3.2.3 Reduction (-N_3_, NO_2_) to Amine

There has been literature evidence in the application of the azide reduction strategy in the development of H_2_S detection probes. Song et al. developed a chromene-based active molecule **15** that showed FL signal enhancement in the red region upon adding H_2_S, with a response time of 6 min.^139^ Further, an economical and portable paper-based device with good reliability and sensitivity for visual monitoring and real-time online analysis of H_2_S was constructed using a smart phone having easy access color-scanning application.^140^ Shen et al. reported the synthesis of a fast response (within 1 min) H_2_S probe **16** through an azide reduction mediated FL detection approach.^24^ The glycosylated quinoline fluorophore endowed the probe with good water solubility to demonstrate its prospective application for the accurate detection of H_2_S in natural water. An ICT based fluorescent sensor **17** for detecting H_2_S ion was developed by Jothi and Iyer.^140^ The efficient reduction of the azide group to amino group transformed the electron withdrawing 1,8-naphthlaimide group into an electron donor, which led to enhanced ICT and “turn-on” FL. With the intention of designing H_2_S sensors with double detection window and red or NIR emission, Xiang et al. fabricated a dual responsive sensor **18** based on fluorophoric dicyanoisophorone dye and H_2_S responsive azide unit.^93^ The red emissive probe demonstrated a relatively large Stokes shift (163 nm) with a double detection window and could respond ratiometrically to HS^–^ for its quantitative sensing in river water at a pH range of 5–8. The amino group of the reaction product underwent protonation to reduce the FL intensity ratio of the probe, limiting its use at lower pH. Moreover, the small molecule probe presented a naked eye detectable color transformation from yellow to pink in the presence of the pollutant.

As rare earth complexes are extremely luminous with high QY and large Stokes shift, Chen et al. constructed a photostable fluorescent europium (Eu) complex **19** with a tripyridine derivative for specific recognition of H_2_S.^141^ The probe had a short response time of 2 min with excellent anti-interference properties and a large Stokes shift for a colorimetric and FL turn-off response for H_2_S. The probe displayed high-precision detection in practical samples based on FL quenching with a reduction in absolute QY from 43.7% to 0.57% in the presence of H_2_S due to the transformation of the electron-withdrawing azide group to electron-donating amino group.

#### 3.2.4 Nucleophilic Substitution

A chromene-based bifunctional trisite coumarin fluorophore **20** was developed by Feng et al. for visualization and quantitative sensing of H_2_S at an optimum pH of 6 in wastewater.^26^ Upon addition of H_2_S, the nucleophilic substitution of -Cl with -SH occurs, and a further intramolecular addition reaction between -CN and -SH produces a 6-membered ring rupturing the π-conjugation system, thereby generating the FL response. The integration of a paper-based sensing platform through a smartphone equipped with a color recognizer app could enable a rapid and cost-effective water quality testing.^4,26^ Saha and group demonstrated the use of probes **21a** and **21b** based on the initial H_2_S-mediated azide-to-amine reduction and bromide-to-thiol nucleophilic substitution, respectively and further cyclization releasing the resorufin fluorophore.^50^ Probe **21a** was more stable and exhibited better sensing in water compared to that of **21b**.

#### 3.2.5 Ligand Exchange/Displacement with Metal Complexes

Generally, the H_2_S probes that rely on reaction-based methods for changes in FL signals largely demand a relatively longer response time which limits their real-life utility.^78,142,143^ The strong hydration tendency of anions to weaken the interaction of the probe with H_2_S poses a challenging situation for the FL sensing of the pollutant thiol in 100% aqueous media.^144,145^ In this context, a metal displacement strategy that relies on the competitive binding of the analyte and a marker to a receptor is an extreme advantage. The displacement sensing mechanism exploiting the strong metal ion affinity of H_2_S can facilitate the attainment of a reaction equilibrium quickly to allow not only rapid response for real-time detection of the biothiol but also the feasible reuse of the receptor. Therefore, a wide variety of small molecule probes based on the metal displacement approach resulting in the formation of metal sulfides has been developed for H_2_S sensing. In addition, fluorescent transition-metal complexes have been constructed recently for specific H_2_S detection in water based on a coordinative approach.

Remarkable water-solubility and striking photophysical features such as narrow emission spectra and long (micro- to millisecond scale) FL lifetimes endow organo-lanthanide complexes with attractive opportunities for time-gated detection of H_2_S. A 1:1 complex was constructed between Cu^2+^ and Eu-complex **22** (emissive) bearing a pyridine-aza-crown motif by Liang and team.^146^ The FL of the water-soluble complex **22** achieved due to the energy transfer facilitated between the pyridine chromophore and coordinated Eu^3+^ ion is quenched by 17-fold upon ligand displacement binding to Cu^2+^. The original Eu emission is restored with a 40-fold FL enhancement in the presence of H_2_S. The highly selective smart FL turn-on gate could offer long-lived Eu emission with a rapid response and binding reversibility to detect H_2_S as low as nanomolar levels in water samples. Hg^2+^ can rapidly react with S^2–^ to form stable HgS with a solubility product of 4 × 10^–53^.^147−149^ Based on this affinity of Hg^2+^ ions to S^2–^, Ma et al. developed a mercuric ion complexed imidazole thione probe **23** that could rapidly react with the metal center to release the ligand resulting in the FL recovery of the system.^28^ The probe immobilized on cellulose acetate paper was used for practical application in detecting H_2_S as low as the 0.7 ppm level. The turn-on/-off FL sensor established good reversible behavior when subjected to H_2_S and Hg^2+^ alternately, which was further used to generate an INHIBIT logic circuit for detecting the two species.

#### 3.2.6 Copper Sulfide (CuS) Precipitation

Several H_2_S sensors based on fast precipitation of CuS due to low solubility (*K*_sp_ = 6.3 × 10^–36^) have been designed. The sensing strategy through CuS precipitation involves two significant practical challenges: to attain adequate selectivity over other anions including thiols and to achieve S^2–^ detection in 100% water media without interference as strong hydration in aqueous media weakens the sensor-S^2–^ interaction.^150^ However, when these challenges are overcome, these reversible luminescence probes are generally attractive due to their low detection limits. Though a variety of Cu(II) centered H_2_S detection including S^2–^ precipitation, chromatography, atomic absorption spectrometry, electrochemical analysis method, etc. have been reported, FL detection being a simple and rapid analysis method is widely used.^151−160^

El-Maghrabey developed a small blue emissive fluorophore **24** with imidazole and pyridine rings, which can coordinate with Cu(II), resulting in FL quenching.^161^ The subsequent restoration of the FL signals upon HS^–^ induced liberation/regeneration of **24** from the coordination complex enables H_2_S detection together with the formation of CuS. The simple synthesis, instant reaction, high-throughput, and miniaturized microplate measurement system is advantageous for H_2_S sensing in environmental water. The technique of using a regeneratable probe and aqueous solvents is also attractive, as they comply with the NEMI quadrant green guidelines and green analytical chemistry principles. Applying a similar concept, Mahnashi et al. designed a fluorimetric sensor **25** that formed a 2:1 complex with Cu^2+^ ions with intense blue FL emission which showed a turn-off response for S^2–^ ion in aqueous media.^162^ Further, based on the water-soluble, amphiphilic, and nontoxic nature of polyvinylpyrrolidone (PVP), a fluorescent cationic probe **26** was prepared by Abd-Elaal et al.^47^ The Cu-**26** secondary probe complex embedded into the PVP structure served as the selective S^2–^ recognition unit, whereas the cationic charge on the polymer surface electrostatically facilitated the S^2–^ and Cu^2+^ ionic interaction resulting in a fast response time (30 s) for S^2–^ detection via turn-off FL in real water samples. A benzimidazole-based fluorescent H_2_S sensor **27** was prepared by Tang et al.^163^ The successive recognition of the **27**-Cu^2+^ complex toward the S^2–^ anion via the Cu^2+^ displacement approach exhibited a quick response and high selectivity at pH 6.0 in 100% water media with good anti-interference ability and FL recovery time within 30 s. Wu et al. reported a regeneratable coumarin–dipicolylamine-based probe **28** for S^2–^ detection in water samples.^23^ The **28**-Cu^2+^ probe displayed a rapid response time with a maximum FL turn-on signal in the presence of 2 equiv of S^2–^ at pH 7.4. The aminoethyl moiety incorporated into dipicolylamine could improve the water solubility and enhance **28**-Cu^2+^ stability in the presence of thiols, thereby increasing the ligand selectivity. Fang et al. synthesized *p*-dimethylaminobenzamide based **29**-Cu^2+^ ensemble as a FL turn-off probe to detect S^2–^ with an LOD value lower than the maximum acceptable concentration in drinking water.^25^ Test paper was used as a convenient and rapid assay for practical application to inspect S^2–^ in real samples, wherein the color of the paper faded, when observed under the naked eye with a turn-off FL response.

Kaushik et al. fabricated a terpyridine based probe **30** and its Cu and Zn complexes for selective H_2_S sensing through turn-on and turn-off FL, respectively.^164^ The metal displacement approach at the terpyridine coordination site facilitated a fast H_2_S detection process (45–60 s) based on the FL signal and could be used for constructing INHIBIT and NAND molecular logic gates. The FL response was faster (within a minute) for the **30**-Cu^2+^ ensemble as opposed to 60 min with **30**-Zn^2+^ at a wide pH range.

Dansyl-based fluorescent probes enable quick and extremely selective identification of target analytes based on PET, FRET, and chelation enhanced fluorescence. In addition, peptides characterized by biocompatibility, good water solubility, and abundant binding sites can be easily obtained from essential amino acids through solid phase peptide synthesis.^165−172^ Based on the concept of combining the advantages of dansyl and peptide as two structural units, Wei et al. prepared a copper peptide backbone based reversible fluorescent probe **31** labeled with dansyl moiety.^173^ The nonfluorescent **31**-Cu^2+^ (AlaHisLys-Cu^2+^) ensemble formed in situ was used as a secondary probe for quick detection of H_2_S via a FL turn-on response, with a much lower LOD compared to that prescribed by WHO and EPA guidelines for drinking water. Moreover, the excellent water solubility of the Dansyl-labeled tripeptide probe **31** was successfully utilized for rapid H_2_S analysis using fluorescent test strips for visual detection.

**Figure 2:**
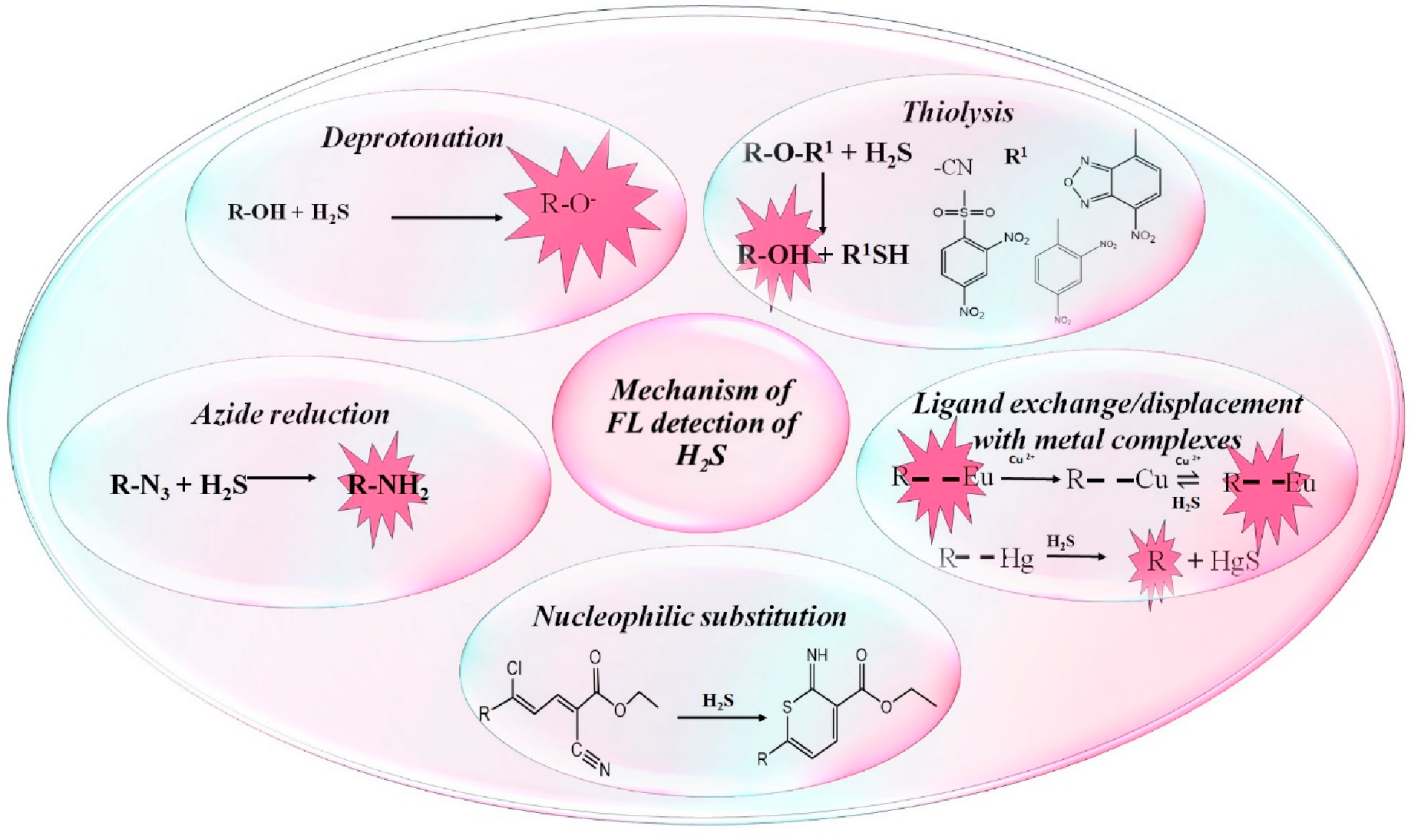
General reaction mechanisms involved in fluorimetric detection of H_2_S using a variety of probes.

The various reaction mechanisms showcased by a variety of probes that enable H_2_S detection in water samples are presented in Figure 2. Moreover, the small molecular probes reported for fluorimetric detection of H_2_S in water samples with the respective detection limits and mechanisms are illustrated in Table 1.

**Table 1:**
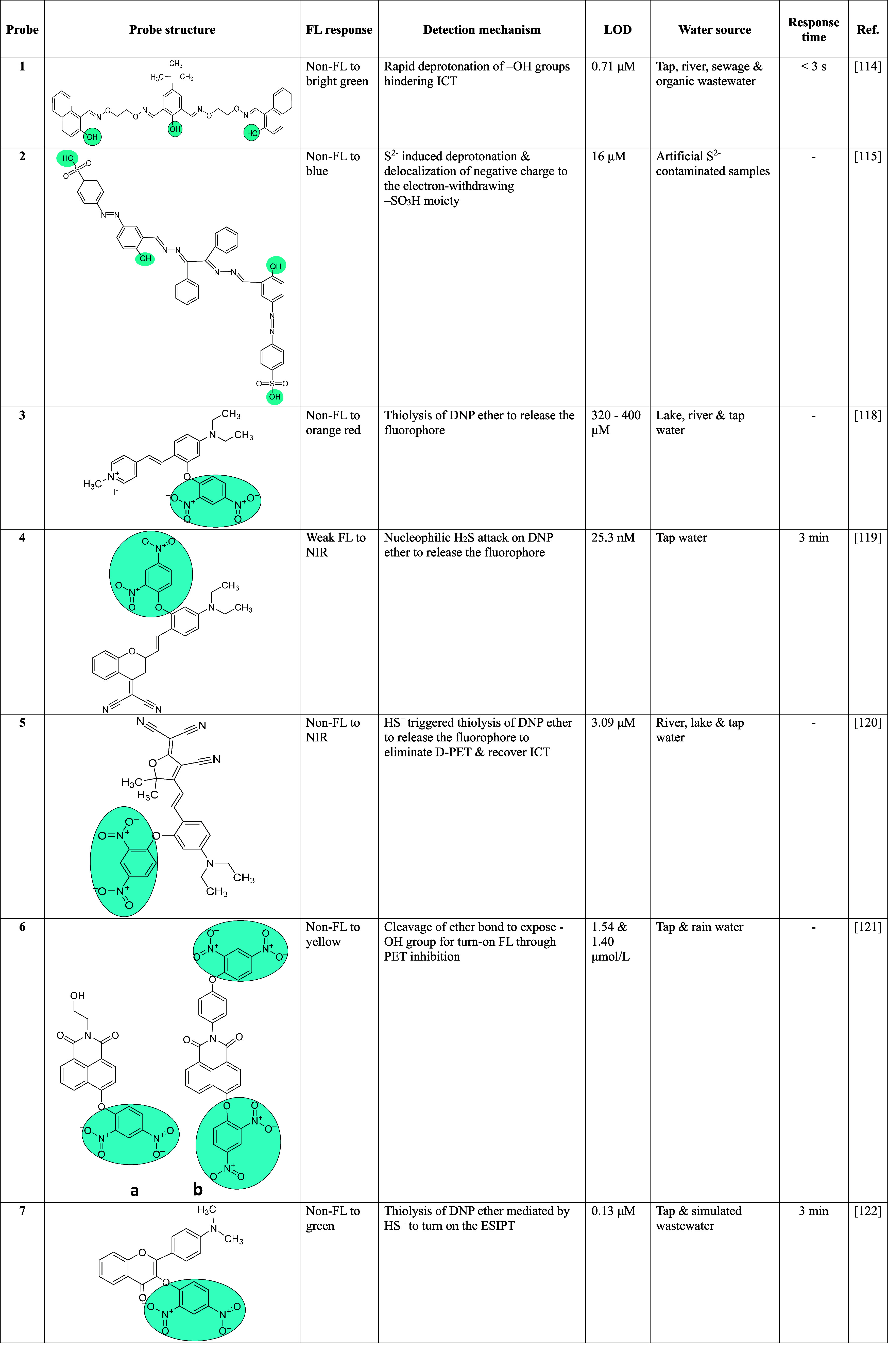

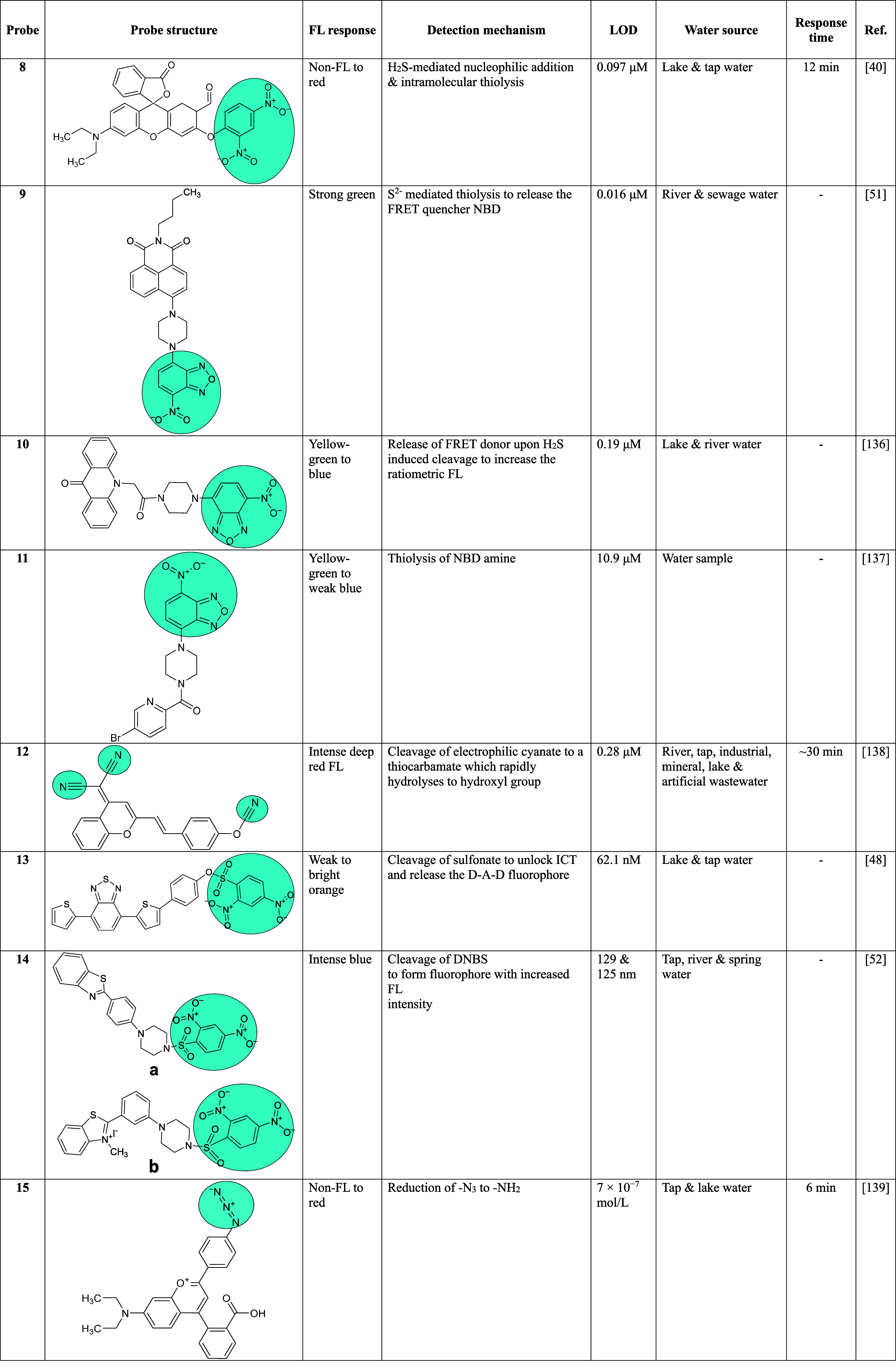

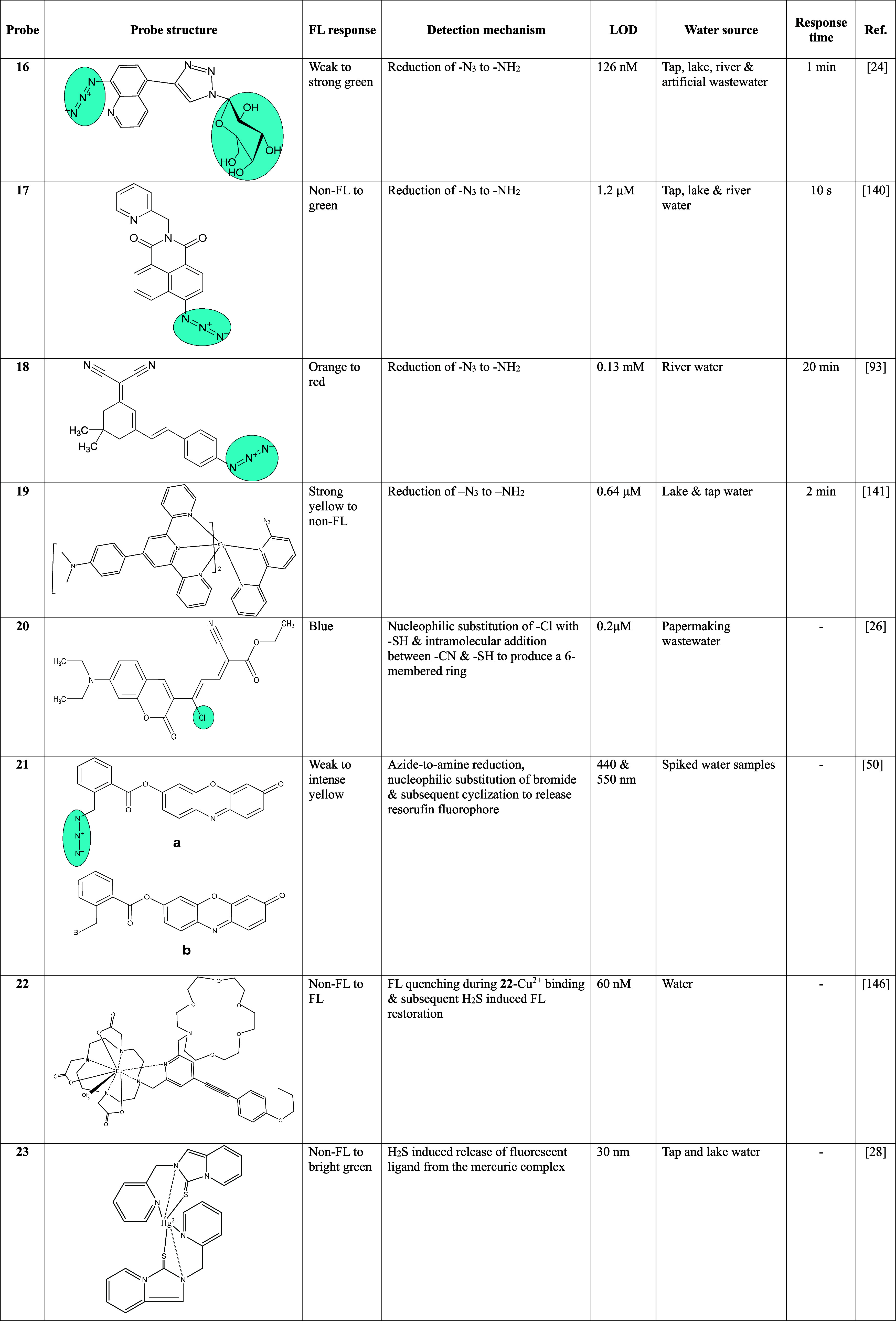

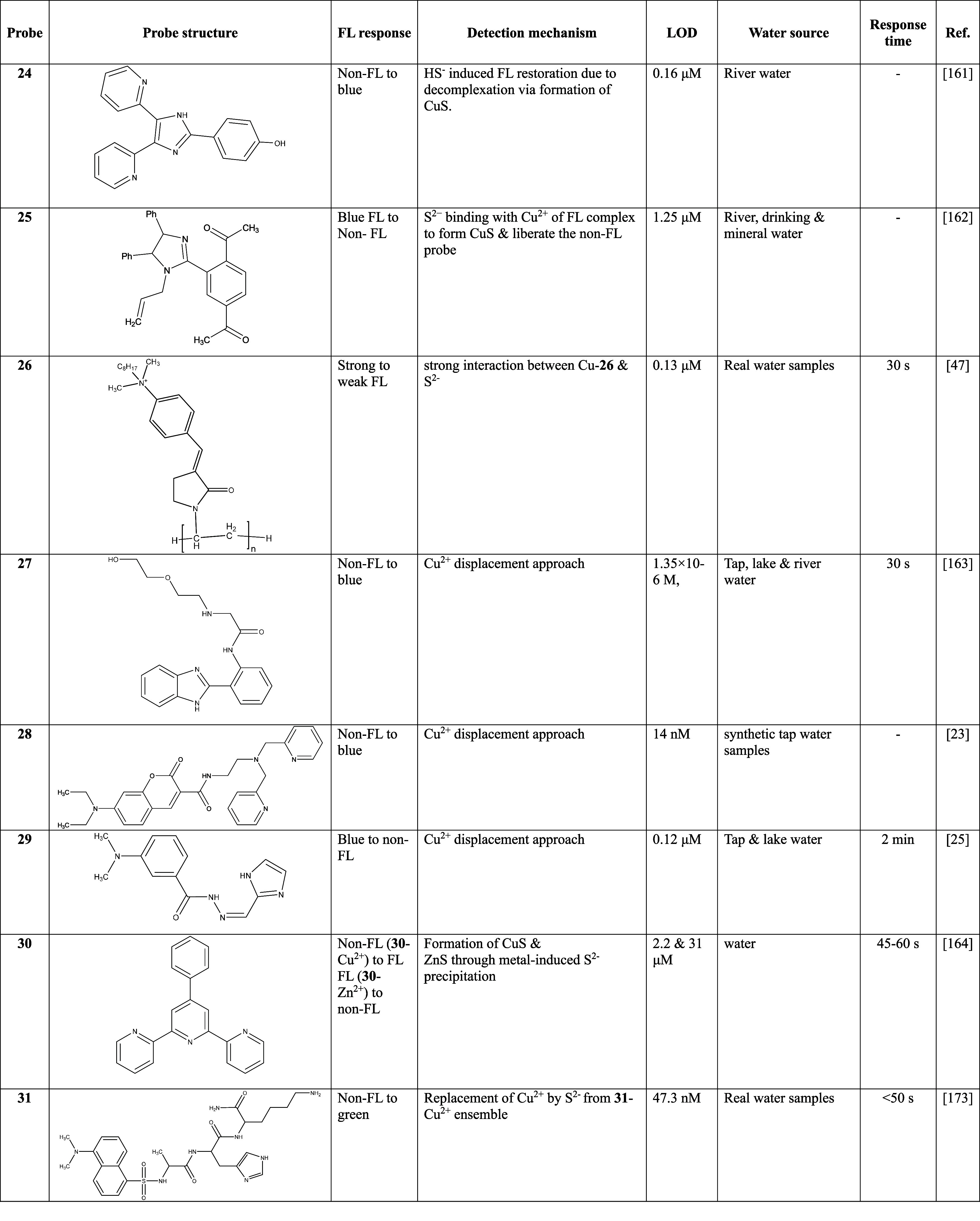
Small Molecule Fluorometric Probes for the Detection of H_2_S in Water Samples

In the reported fluorescent probe based on the deprotonation mechanism, the reversibility of the reaction that allows their reuse is an advantage, especially notable for probes that exhibit a fluorescence based and visually observable color transition triggered by sulfide ions. This feature holds potential for practical applications including the development of molecular logic gates. Conversely, probes utilizing thiolysis offer various advantages, such as easy synthesis, visible light excitability, and rapid detection within minutes. These probes provide dual colorimetric and fluorimetric responses, along with high selectivity and sensitivity toward H_2_S. Additionally, the nucleophilic addition of H_2_S to the aldehyde group in certain probes induces intramolecular thiolysis, resulting in a rapid fluorescence turn-on response. The cleavage of the DNBS group leads to a significant increase in fluorescence intensity, while the cleavage of the sulfonamide group triggers a NIR fluorescence response in the presence of H_2_S. In the case of the reduction-based mechanism, the probe demonstrates high-precision detection in practical samples, attributed to turn-on fluorescence resulting from the transformation of the electron-withdrawing -N_3_, NO_2_ groups to an electron-donating amino group. During nucleophilic substitution, probe **21a** exhibits better stability and sensing capabilities in water compared to those of **21b**. However, probe **21b** displays a faster response time due to solvolysis. In probes utilizing ligand exchange/displacement with metal complexes, the remarkable water solubility and photophysical features of organo-lanthanide complexes enable time-gated detection of H_2_S. On the other hand, probes based on CuS precipitation face challenges in achieving adequate selectivity in detecting S^2–^ in 100% water media without interference from other anions. Therefore, the most effective probes are those with the lowest LOD and rapid response times (in seconds), regardless of the detection mechanism employed.

## 4 Small Molecules as Colorimetric H_2_S Probes

Fluorimetric detection, as mentioned in the previous section, involves the need for using a fluorimeter to quantitatively measure the fluorescence changes in response to H_2_S in the analyte. However, colorimetric visual detection systems are popular and highly attractive because of their ability to rapidly sense the analyte with the naked eye and avoid the need for any expensive instrumentation.^174,175^ These simple and portable sensors that exhibit naked eye signals can be readily fabricated with minimal cost for in situ or in the field detection of environmentally important analytes including H_2_S. A spectrophotometer or other straightforward device can also be used to measure the color shift and quantify the analyte. Some H_2_S sensors that depend on the optical changes of reagents immobilized on solid supports are also developed. Among these, paper loaded with probes have achieved significant impact in analytical research due to their intrinsic chemical features including molecular structure, chemical functionalizability, low-cost, varying thickness possibility, porosity, high mechanical flexibility, ability to be infused with liquid/water samples through capillary action and further hold them, printable with sensing agents, and easily viewable color changes.^176^ These smart paper-based sensors can be used in the colorimetric detection of HS^–^ and S^2–^ in water media. Moreover, quantitative detection of these ions based on their concentrations is also possible by measuring the strength of the color imparted to the strip. These strips that can reveal naked eye detectable color transformations can be exploited as portable testing kits for spot analysis of H_2_S/HS^–^/S^2–^ content in water samples collected from diverse sources.

**Figure 3:**
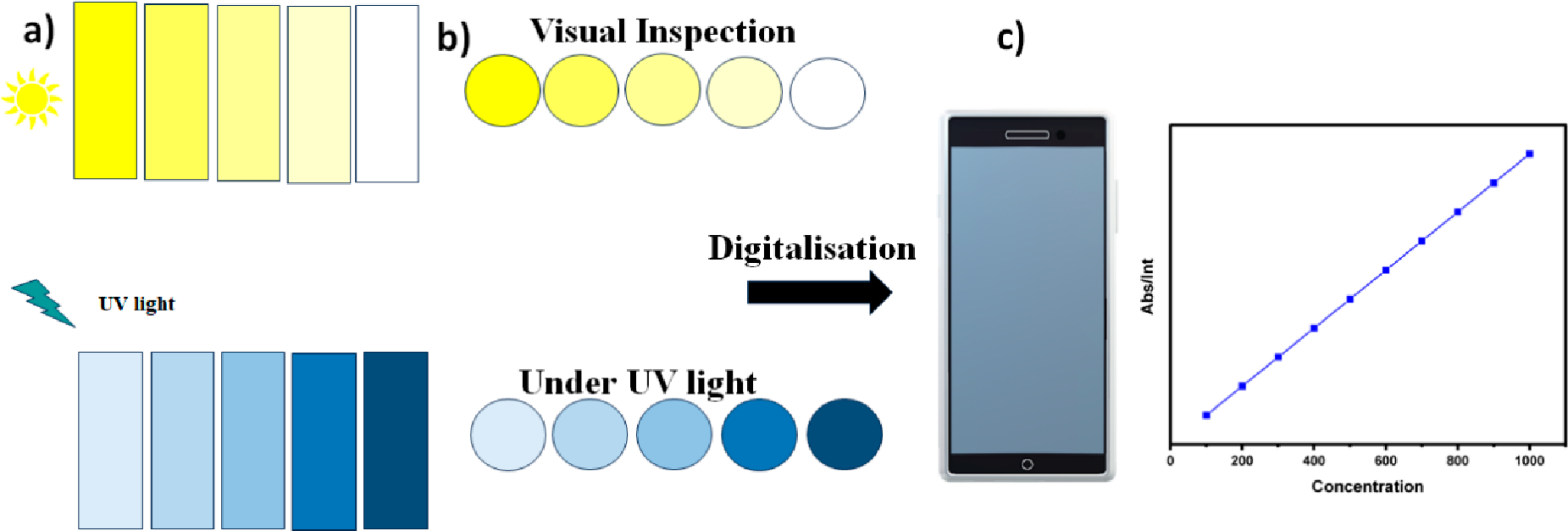
H_2_S detection (a) in water samples and (b) on paper strips colorimetrically and fluorimetrically. (c) Digitalization of data allowing in situ quantification of H_2_S levels.

The advancements in smartphone technologies also unlock innovative and captivating avenues to improvise optical analytical tools. The paper-based devices can be equipped with different gadgets that integrate high resolution cameras and powerful processors with high storage capacity.^177,178^ Moreover, image-editing openly available software is also complementary facilitators for scientific improvements in these sensing devices.^31^ Besides, the data obtained after in situ field testing of samples can be immediately shared through Internet connectivity using smartphones.^177^ Fluorimetric and colorimetric techniques can be used together to detect and quantify the pollutant in water samples to obtain in situ data generation. Figure 3 depicts the fluorimetric and colorimetric detection of H_2_S in water samples and on paper-based strips, and subsequent in situ analysis using a digitalized technique.

### 4.1 Design Strategy for Colorimetric H_2_S Probes

The most common strategy is the use of a chemodosimeter, wherein a H_2_S selective unit and an optical signaling response modulator is judiciously incorporated into the probe. The probes are categorized into two based on the detection mechanism: (i) “reactive” probes that work on irreversible S^2–^-specific chemical reactions centered on the double nucleophilic nature and reduction ability of H_2_S and (ii) “competitive” probes that utilize displacement of metal ions to selectively react with H_2_S and not with any other sulfur containing species or anions. Nitro, hydroxylamine, azide,^179−189^ DNP ethers,^189−197^ nitrobenzoxadiazole (NBO) ether,^197,198^ and NBD^199−203^ are the copiously used functional groups for the colorimetric detection of H_2_S.

### 4.2 Colorimetric Sensors Based on Various Reaction Mechanisms

The small molecule H_2_S sensors that are reported solely on colorimetric signals are reviewed below.

#### 4.2.1 Thiolysis Reactions

Jothi et al. synthesized a phenanthridine derivative **32** incorporated with H_2_S selective DNBS group as signaling unit.^204^ Thiolysis of **32** initiated a pronounced visual color development to dark yellow with a fast response in <10 s. The filter papers coated with probe **32** can be used for onsite qualitative testing of H_2_S. Das and Sahoo developed a dansyl-naphthalimide conjugated sulfonamide probe **33** for selective and rapid H_2_S detection in environmental samples.^205^ H_2_S/HS^–^ reacts with the sulfonamide center of DNPS (SNAr pathway) to generate naphthalimide hydrazone, SO_2_, and dansyl thiol. The dark purple color developed due to the generation of dansyl thiol facilitates the colorimetric sensing of H_2_S in water in the pH range of 7–8.

#### 4.2.2 Deprotonation Process

Due to the tendency to form strong hydrogen bonds, S^2–^ probes that contain acidic NH and OH groups can be constructed. Ryu et al. designed a colorimetric probe **34** through a deprotonation process for selective H_2_S detection over 6–11 pH range to induce a pale yellow to pink color change over most other competitive ions in aqueous solution.^206^

#### 4.2.3 Metal Sulfide Precipitation

Nitrogen and sulfur can serve as suitable metal ion binding sites to facilitate colorimetric sensing of H_2_S based on a metal displacement approach.^207,208^ The strong affinity of S^2–^ for transition metal ions including Cu^2+^ (*K*_sp_ for CuS = 6310–36) and Hg^2+^ (*K*_sp_ for HgS = 2310–53) is advantageous to develop probes with a fast response time and reversibility.^209−212^ Choe and Kim constructed a colorimetric H_2_S complex probe **35** based on benzothiadiazole as the A group and julolidine as the D unit.^213^ The violet colored **35**-Cu^2+^ chromogenic chemosensor and coated test strips could detect H_2_S in real water samples via a cation displacement process. A probe with a charge donating chromophoric unit and adjacent thiol and amine functionalities as the binding site could serve as an efficient receptor for Hg^2+^ binding. Hence, Kaushik et al. constructed a Dabsyl based 2-aminothiophenol sensor **36** for selective colorimetric detection of H_2_S in water medium among various biothiols and anions.^209^ The **36**-Hg^2+^ ensemble based on the Hg^2+^ displacement approach displayed a notable visual detection of H_2_S.

#### 4.2.4 Methylene Blue Detection

Pla-Tolós et al. prepared a low-cost colorimetric sensor **37** based on the immobilization of *N*,*N*-dimethyl-*p*-phenylenediamine and ferric chloride on cellulose paper support.^214^ The adsorption of H_2_S on the probe coated paper sensor undergoes a reaction to generate highly stable methylene blue. The characteristic blue color developed due to the formation of the thiazine dye could provide accurate and precise results as the method not only evades the preparation of derivatization reagents and sample treatment but also enables in situ measurements. The paper-based sensing can be combined with mobile phones to build a smart and practical analytical method to detect H_2_S in water. Table 2 lists the small molecular probes reported for the colorimetric detection of H_2_S in water samples with the respective detection limits and mechanisms.

**Table 2:**
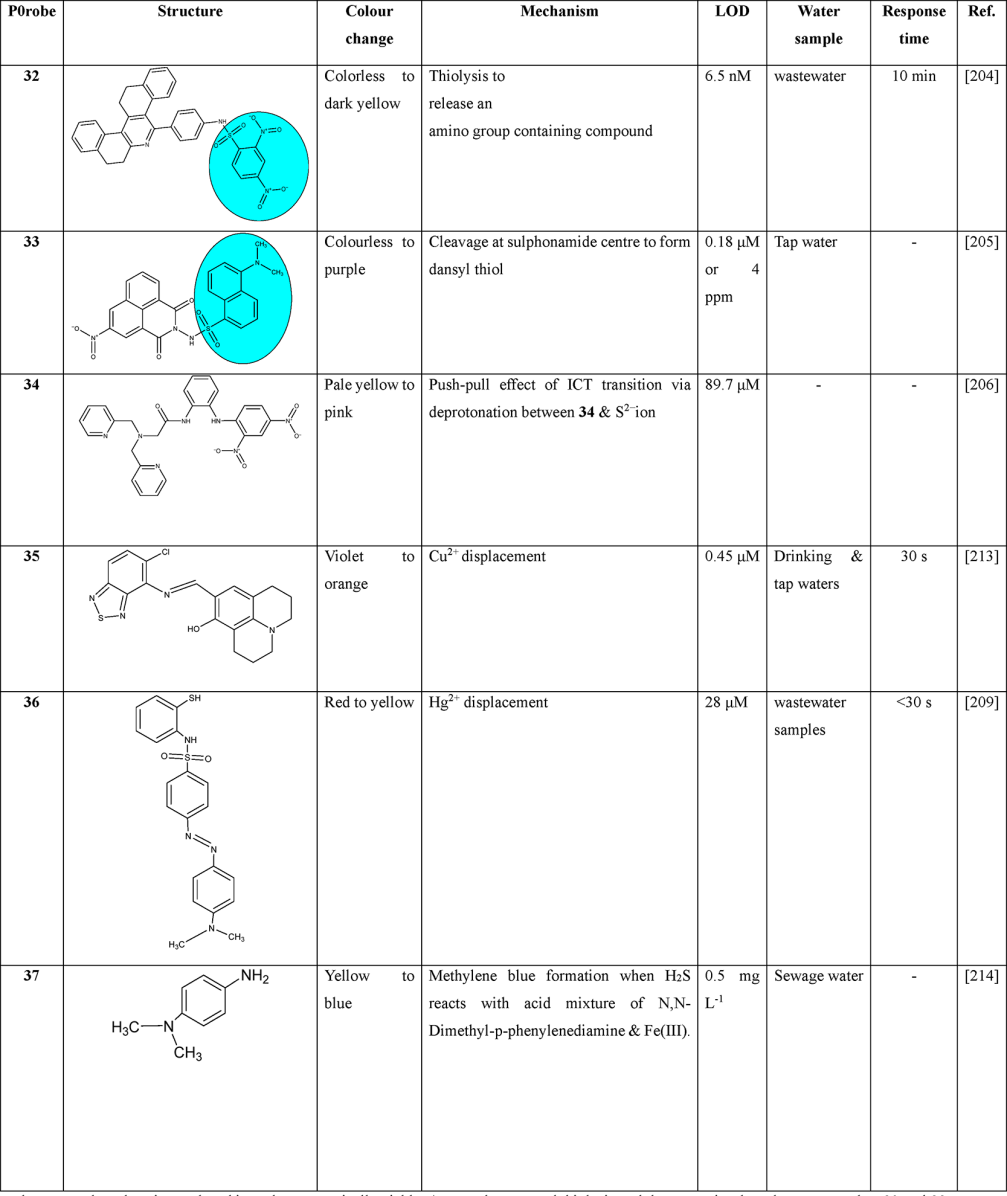
Small Molecule Colorimetric Sensors for H_2_S in Water

Probe **32** under a thiolysis reaction have shown a remarkable response time (10 s) and responded to H_2_S levels as low as 6.5 nM. Its real-time application such as paper-based testing makes this probe economically viable. Among the reported thiolysis and deprotonation-based sensors, probes **32** and **33** are more attractive due to their very quick response time with a much lower detection limit and real time application. Probe **35** detects H_2_S based on the Cu^2+^ displacement approach with a fast response time of 30 s and an LOD of 0.45 μM compared to the Hg^2+^ displacement reaction to sense H_2_S with a higher detection limit of 28 μM. It is observed from the literature evidence that metal sulfide precipitation displayed a faster response time and reversibility.

## 5 Limitations and Future Perspectives

As optical sensors are noninvasive, highly sensitive, and selective, they have a wide range of applications in a variety of industries for H_2_S detection. These sensors are essential in industrial environments, especially in sectors like oil and gas, chemical production, and wastewater treatment, where H_2_S is frequently produced as a byproduct. Early detection of leaks can reduce the danger of exposure to toxic quantities of the gas, help prevent accidents, and guarantee worker safety. It is possible to measure the amounts of H_2_S in air and water by using optical sensors. The food sector and health care are other fields in which H_2_S sensors have translational applications. During emergency situations, portable and fast-responding optical sensors for H_2_S detection can be utilized, including chemical spills or mishaps. Furthermore, optical sensors are effective tools in lab research for investigating H_2_S related processes in chemistry, biology, and environmental science. These probes function as excellent instruments for a variety of practical and scientific uses, and their translational applications help to improve safety, environmental monitoring, and quality control across several industries.

Though H_2_S detection through changes in FL signals has been extensively demonstrated to be advantageous due to moderate to rapid response, low limit of detection, and low to moderate selectivity, targeting capability of these fluorophores still needs to be improved to realize their applications in aqueous media. Moreover, low background interference, long-wavelength FL, and a large Stokes shift are the emission features that are always sought after in fluorimetric H_2_S probes. Therefore, the quest for a competent fluorescent probe suitable for detecting H_2_S in environmental systems is still on. Practical applicability of the probe with a rapid response time is yet another vital factor for in situ detection of H_2_S quantitatively. A combination of highly selective and sensitive FL assays coupled with high throughput, low cost, and time effective assays can benefit the real life in situ detection of H_2_S. Further, many probes that rely on the commonly used azide reduction approach suffer from complex synthetic procedures and/or long response time of ∼0.5 to 2 h to obtain maximum signal changes. Besides, the azido-fluorophores are generally photolabile to produce fluorescent amino-fluorophores.^214^ Moreover, most of the H_2_S FL probes use UV light (<400 nm) as an excitation source—a drawback for applications where visible light excitation source is used. Hence, probes that can be excited using visible light are anticipated to be developed.

There exists a pressing need to construct newer small molecules as H_2_S probes with improved features. Many of the fluorometric detection methods use environmentally harmful organic solvents or toxic metals. Selective H_2_S sensors that can be used in 100% aqueous media are scarce.^29,121,215,216^ Hence, the design and construction of simple and effective small molecular systems that can be applied in water samples are still imperative. Moreover, most of the dual roles of H_2_S detectors reported are effective only in organic solutions, restricting their applicability in aqueous media. Consequently, developing a dual strategy for selective and fast sensing of S^2–^ ion in aqueous solutions is essential for environmental wellness. The structural modification of small molecule probes to accommodate hydrophilic functional groups or positive charge to improve their water solubility can be explored.

Among the major approaches, S^2–^-specific chemical reactions based H_2_S sensors have gained wide attention. However, FL probes that work on azide reduction and nucleophilic addition of the S^2–^ ion are largely irreversible and mostly need considerably long reaction duration. Therefore, the design and construction of reversible FL probes for real time quantification of H_2_S might be more beneficial. Further, FRET mechanism based H_2_S probes have severe drawbacks due to inherent water solubility issues, low sensitivity attributed to lower FL QY, and significant cross sensitivity for sulfite ions during S^2–^ sensing. In addition, though few probes based on the FRET strategy are developed, the majority of them are FL turn-on sensors, whereas only a few are ratiometric sensors. Hence the design of small molecules as ratiometric probes needs to be further explored.

Colorimetric sensors have garnered considerable research attention, as they offer instant visually detectable color switches to detect analytes. These valuable traits intrinsic to chromogenic probes can facilitate monitoring of H_2_S emission levels in various industrial platforms and target environments. Nevertheless, research on the advancement of selective and sensitive colorimetric sensors for H_2_S is not sufficient. Though a few colorimetric H_2_S sensors have been reported in the recent past, selectivity over competing biothiols and anions, in addition to response time, is a serious limitation. Among the various H_2_S receptors, development of colorimetric sensors is more complicated than the others because of pH sensitivity, interaction of counterions, and more hydration. Furthermore, highly sensitive H_2_S probes that rely on an anion induced deprotonation mechanism in 100% water and probes with multiple recognition sites are still rare.

Paper-based analytics are one of the most cost-effective detection platforms for remote field assays. The sensitivity of paper-based tests can be significantly improved by employing a test strip reader, a hand-held colorimeter, or a smartphone to quantitatively measure H_2_S induced change in color intensity. Through this study, we expect to provide insights into the present status of sensors developed for H_2_S detection in water and further research efforts that are required to explore the design and construction of H_2_S probes, which can overcome the above-mentioned limitations for in situ real life applications.

## List of Acronyms

A: Acceptor
CN^–^: Cyanide
Cu: Copper
CuS: Copper sulfide
D: Donor
DNP: Dinitrophenyl
DNBS: Dinitrobenzenesulfonate
ESIPT: Excited-state intramolecular proton transfer
Eu: Europium
FL: Fluorescence
FRET: Fluorescence resonance energy transfer
Hg: Mercury
H_2_S: Hydrogen sulfide
HS^–^: Monohydrogen sulfide
ICT: Intramolecular charge transfer
LOD: Limit of detection
NBD: 7-Nitro-1,2,3-benzoxadiazole
NIR: Near-infrared
PET: Photo induced electron transfer
PVP: Polyvinylpyrrolidone
QY: Quantum yield
S^2–^: Sulfide
